# Age-Related Expansion of Tim-3 Expressing T Cells in Vertically HIV-1 Infected Children

**DOI:** 10.1371/journal.pone.0045733

**Published:** 2012-09-24

**Authors:** Ravi Tandon, Maria T. M. Giret, Devi SenGupta, Vanessa A. York, Andrew A. Wiznia, Michael G. Rosenberg, Esper G. Kallas, Lishomwa C. Ndhlovu, Douglas F. Nixon

**Affiliations:** 1 Hawaii Center for AIDS, Department of Tropical Medicine, John A. Burns School of Medicine, University of Hawaii, Honolulu, Hawaii, United States of America; 2 Division of Experimental Medicine, University of California San Francisco, San Francisco, California, United States of America; 3 Division of Clinical Immunology and Allergy, University of São Paulo, São Paulo, Brazil; 4 Albert Einstein College of Medicine, Bronx, New York, United States of America; Massachusetts General Hospital, United States of America

## Abstract

As perinatally HIV-1-infected children grow into adolescents and young adults, they are increasingly burdened with the long-term consequences of chronic HIV-1 infection, with long-term morbidity due to inadequate immunity. In progressive HIV-1 infection in horizontally infected adults, inflammation, T cell activation, and perturbed T cell differentiation lead to an “immune exhaustion”, with decline in T cell effector functions. T effector cells develop an increased expression of CD57 and loss of CD28, with an increase in co-inhibitory receptors such as PD-1 and Tim-3. Very little is known about HIV-1 induced T cell dysfunction in vertical infection. In two perinatally antiretroviral drug treated HIV-1-infected groups with median ages of 11.2 yr and 18.5 yr, matched for viral load, we found no difference in the proportion of senescent CD28^−^CD57^+^CD8^+^ T cells between the groups. However, the frequency of Tim-3^+^CD8^+^ and Tim-3^+^CD4^+^ exhausted T cells, but not PD-1^+^ T cells, was significantly increased in the adolescents with longer duration of infection compared to the children with shorter duration of HIV-1 infection. PD-1^+^CD8^+^ T cells were directly associated with T cell immune activation in children. The frequency of Tim-3^+^CD8^+^ T cells positively correlated with HIV-1 plasma viral load in the adolescents but not in the children. These data suggest that Tim-3 upregulation was driven by both HIV-1 viral replication and increased age, whereas PD-1 expression is associated with immune activation. These findings also suggest that the Tim-3 immune exhaustion phenotype rather than PD-1 or senescent cells plays an important role in age-related T cell dysfunction in perinatal HIV-1 infection. Targeting Tim-3 may serve as a novel therapeutic approach to improve immune control of virus replication and mitigate age related T cell exhaustion.

## Introduction

Since the advent of antiretroviral drugs, perinatally HIV-1-infected children have grown up into the adolescent age with lower rates of AIDS related mortality and morbidity [1], [2], [3], [4], [5]. Despite combination antiretroviral therapy (cART), perinatally HIV-1 infected subjects have striking differences in HIV-1 disease progression compared to adults and adolescents and have higher viral load (VL) and lower virological responses rates than adults [6], [7], [8], [9]. This is primarily as a consequence of poor adherence to drugs over a lifetime, underdosing, treatment fatigue, altered pharmacokinetics, novel toxicities, caregiver-related problems and high rates of psychiatric illness including the complications of long-standing infection and the deleterious effects of cART [6], [10], [11], [12]. In horizontally infected adults with chronic treated HIV-1 infection, it is evident that mortality due to non-AIDS events is more common than mortality due to AIDS-related events [13] and this could potentially occur in perinatally infected children earlier. As perinatally HIV-1-infected children age with HIV-1, deleterious consequences to protective T cell immunity may persist or develop despite cART [6], [14]. The exact nature of these immunological events and the association with disease progression in vertically infected patients remain unclear.

On encountering antigen, CD8^+^ T cells differentiate from the least differentiated (naive or early memory) stage to the most mature (memory/effector) stage. In this process, cell surface receptors are progressively downregulated (CD45RA, CCR7, CD28, CD27, CD127) or upregulated (CD57 and CD45RA) as CD8^+^ T cells differentiate [15], [16], [17], [18]. In adults with HIV-1 infection, T cells fail to fully mature into effector T cells [19], [20], [21]. We have previously shown that the differentiation status of HIV-1 specific T cells in adults were not readily altered by cART despite declines in T cell activation suggesting that cART does not reverse T cell effector defects [14]. We further showed that in perinatally infected children, T cell effector maturation induced by HIV-1 infection was markedly weaker compared to adults, even in those on cART [22]. As HIV-1 specific T cells develop increased CD57 expression, they have replicative senescence [23], and remain senescent despite suppressive cART.

During many chronic viral infections a distinct terminal state of T cell differentiation, or T cell exhaustion arises [24], [25]. Such functionally impaired T cells are characterized by abnormally low cytokine production, poor proliferative capacity with the upregulation of several inhibitory receptors including Programmed Death-1 (PD-1) and T-cell immunoglobulin and mucin domain-containing molecule-3 (Tim-3) among others [26], [27], [28], [29]. These receptors not only mark but also induce inhibitory signals to dampen T cell immune responses. In HIV-1 infection, PD-1, a CD28 family member, is increased on CD8^+^ T cells in progressive HIV-1 disease [30], [31]. Tim-3, an immunoglobulin (Ig) superfamily member, initially identified as a negative regulator of Th1 response through the Tim-3/Galectin-9 pathway in several inflammatory disease states [32], [33], is also elevated in HIV-1 disease [30] and associated with disease progression. cART can reduce PD-1 levels in T cells in HIV-1 infected adults, and in some subjects Tim-3 expression is also reduced [30], [34]. Several combinations of markers are therefore used to discriminate differentiated and senescent T cells. However, whether there are common mechanisms regulating them remain unclear [35].

In the setting of treated HIV-1 disease in adults, T cell function remains perturbed with CD8^+^ T cell activation, defined by CD38^+^ or CD38^+^HLA-DR^+^ coexpression, at higher levels than in uninfected subjects [14], [36], [37]. Immune activation occurs in perinatally infected children and the degree of immune activation present as early as 1–2 months of age can be used to predict which children will become long-term non-progressors [38], [39]. In children, the relationship between HIV-1 viral load and immune activation appears to be less clear-cut than in adults, in whom a higher level of viremia is predictably associated with higher levels of activation [38], [39], [40]

As adults with HIV-1 infection age, it appears that alterations in immune profile or immunosenescence begin to resemble those of much older uninfected subjects, and is now referred to as “premature aging” [41], [42], [43]. Phenotypic and functional T cell alterations observed during advancing human age lead to poor responses to and efficacy of vaccines, and increased susceptibility to new infections and tumors in the elderly [44], [45]. With an acceleration of aging and T cell decline induced by HIV-1 infection, a similar impact on T cell immunity could occur in perinatal HIV-1 infected children as they age into adolescenthood.

In this study we sought to assess the effects of HIV-1 infection on T cell differentiation, senescence and exhaustion in two age groups of perinatally HIV-1-infected subjects with shorter and longer durations of infection that present with persistently high HIV viremia despite access to cART, and determine the associations with markers for disease progression (viral load, immune activation) to improve our understanding of how these parameters may modulate the ability of protective T cell immunity to control HIV infection in children.

## Materials and Methods

### Ethics Statement

The research involving human participants reported in this study was approved by University of California, San Francisco (UCSF) and Albert Einstein College of Medicine (AECOM) institutional review boards (IRBs), with the approval numbers 10-04893 (UCSF) and 1999-255-000 (AECOM). The legal guardians (biological parent, adoptive parent etc.) provided written informed consent for these patients. For children above 12 years of age, signed consent on the regular informed consent document along with their legal guardians was obtained; for children between the ages of 7 through 12, child assent was also required and obtained; for children less than 7 years old, only the legal guardian provided written consent. The research was conducted according to the Declaration of Helsinki.

### Study Population

The study population included 16 perinatally HIV-infected subjects from the Jacobi Medical Center, Bronx, New York. Whole blood samples were collected in EDTA tubes from the subjects during their scheduled monthly visit after obtaining informed consents. Peripheral blood mononuclear cells (PBMC) were purified using Ficoll-Paque™ PLUS density gradient centrifugation (Amersham Pharmacia Biotech, Uppsala, Sweden). Cells were frozen in media containing 90% fetal bovine serum (HyClone, Logan, UT) and 10% dimethyl sulfoxide (Sigma Aldrich, St. Louis, MO) and stored in liquid nitrogen. All subjects were under the care of pediatricians at the Jacobi Medical Center, Bronx, New York.

### Measurement of Viral Load

Plasma HIV-1 viral load (VL) was measured with Amplicor HIV-1 Monitor with a lower limit of detection of 50 copies of RNA/ml (Roche Diagnostic Systems, Branchburg, NJ).

### Flow Cytometry Assessment

Cryopreserved PBMC were thawed in 37°C water bath, washed in RPMI-1640 medium (HyClone, Logan, UT) supplemented with 10% fetal bovine serum. The PBMC were used in two different panels for surface staining. Briefly, 1×10^6^ PBMC were washed in FACS buffer (PBS+0.02% EDTA and 1% BSA) and transferred to a 96-well V-bottom plate, surface stained for different surface markers for 30 minutes on ice followed by washing in FACS buffer twice and then fixing in 1% paraformaldehyde (Polysciences, Niles, IL) on ice. Finally, cells were analyzed on a LSRII flow cytometer (Becton Dickinson, San Jose, CA). The data was analyzed with FlowJo software, version 9.0 (Tree Star, Ashland, OR). Panel 1 included PE-anti-CD38 (BD Biosciences, San Jose, CA), FITC-anti-HLA-DR (BD Biosciences), Alexa 700-anti-CD4 (BD Biosciences), Qdot 605-anti-CD8 (Invitrogen, Carlsbad, CA), ECD-anti-CD3 (Beckman Coulter, Brea, CA). Panel 2 had PE-anti-Tim-3 (R&D System, Minneapolis, MN) APC-anti-PD-1 (Biolegend, San Diego, CA), Alexa 700-anti-CD4 (BD Biosciences), ECD-anti-CD3 (Beckman Coulter), APC Cy7-anti-CD8 (BD Biosciences), PE-Cy7-anti-CD28 (eBiosciences, San Diego, CA), and FITC-anti-CD57 (BD Biosciences). An amine aqua dye (Invitrogen) was also included in both panels to discriminate between live and dead cells.

### Statistical analysis

Statistical analysis was performed using GraphPad Prism statistical software (GraphPad Software, San Diego, CA). The nonparametric Mann-Whitney U was used for comparison tests, and the Spearman Rank test was used for correlation analyses.

## Results

### Human Subjects

Sixteen HIV-1-infected pediatric subjects of Hispanic and Black ethnicity were divided into two groups based on age. The median age of the two groups consisting children (n = 6) and adolescents (n = 10) was 11.2 and 18.5 years, respectively (Table 1). The two groups were matched for viral load and CD4 count (Table 1). All except three subjects in adolescents were on cART with variable adherence to antiretroviral treatment. The characteristics of both groups are displayed in table 2.

**Table 1:** Numbers, CD4 count, viral load, CD8+ T cell activation, and age of subjects in each group.

Measurement	Children	Adolescents	P-value
N	6	10	
Median CD4^+^T cell count (cells/mm^3^ [IQR])	547 (299–1,223)	380.5 (338.5–426.5)	0.41
Median HIV-1 viral load (log10 c/ml [IQR])	3.8 (2.8–4.7)	4.5 (4.0–4.8)	0.18
Median CD8^+^ T cell activation (% CD38, HLA-DR [IQR])	33.05 (19.95–37.98)	16.15 (16.17–29.63)	0.103
Median age (yr [IQR])	11.2 (11.0–11.8)	18.5 (17.4–19.9)	0.0002*

**Table 2:** Subject characteristics.

Group	Patient ID	Age (in yrs)	R/E*	Sex	ART§	Notes
*Children*	S1	11.0	W/H	M	ABC, 3TC, ATV/r	-
	S2	11.0	B/NH	F	ddI, 3TC, LPV/r	Variable adherence.
	S3	11.1	W/H	M	d4T, EFV, LPV/r	Excellent adherence.
	S4	11.4	B/H	F	ABC, 3TC, ATV/r	-
	S5	11.8	W/H	F	ZDV, 3TC	Long term PTI patient.
	S6	12.0	B/NH	F	ZDV, 3TC	-
*Adolescents*	P1	19.3	W/H	M	On etravirine study	Complete non-adherence to ART.
	P2	19.6	W/H	F	ZDV, 3TC, LPV/r	2nd trimester pregnancy, improved adherence.
	P3	21.2	B/NH	M	EFV-based HAART	Variable adherence.
	P4	22.8	W/H	M	TDF, FTC, ddI, ATV	-
	P5	18.5	W/H	M	ZDV, 3TC	-
	P6	18.6	W/H	F	No ARVs	Off ARVs for >2 yrs.
	P7	18.0	B/NH	F	No ARVs	AIDS, HIV nephropathy, on hemodialysis, non-adherence.
	P8	17.7	W/H	F	ZDV, 3TC, ABC, TDF, ATV/r	Complete non-adherence to ART.
	P9	16.5	W/H	M	No ARVs	Off ARVs for >2 yrs.
	P10	16.3	W/H	F	ZDV, 3TC, ABC, RTV	Poor adherence.

R/E = Race/Ethnicity, W/H = White/Hispanic, B/NH = Black/Non-Hispanic,

§ ART = Antiretroviral treatment, ZDV = Zidovudine, 3TC = Lamivudine (2′, 3′-dideoxy-3′-thiacytidine), ABC = Abcavir, TDF = Tenofovir disoproxil fumarate, ATV/r = Atazanavir/ritonavir, LPV/r = Lopinavir/ritonavir, ddI = Didanosine, EFV = Efavirenz.

### There are similar proportions of CD28^−^/CD57^+^ T cells in children and adolescents

**Figure 1:**
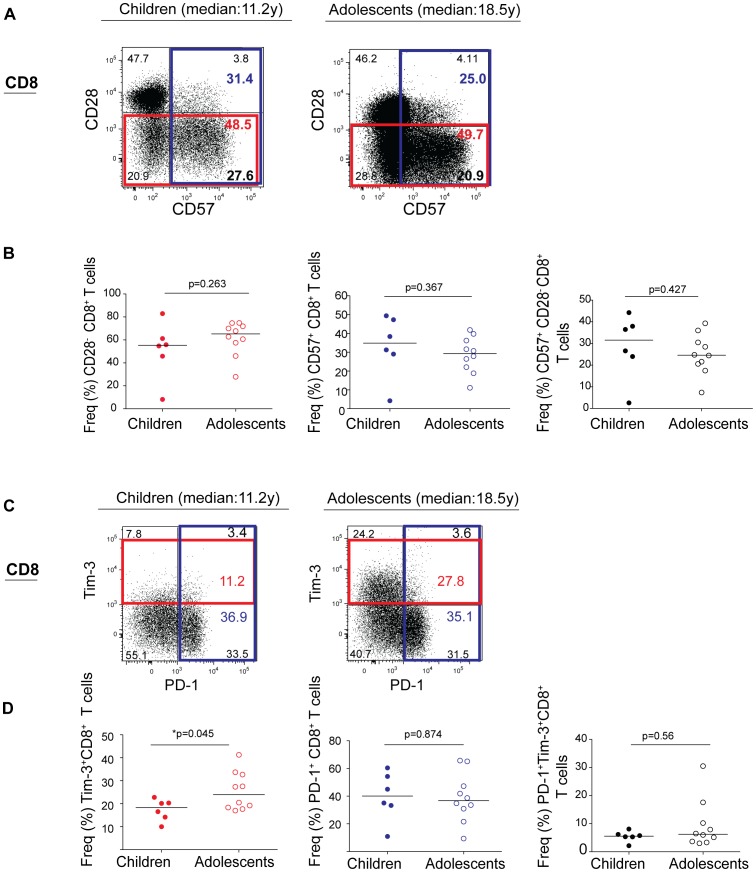
Expansion of Tim-3^+^CD8^+^ T cells in perinatally HIV-1-infected adolescents. (A) Flow dot plots show CD8^+^ T cell surface expression of CD28 and CD57 in perinatally HIV-1-infected children with shorter duration of HIV-1 infection (median: 11.2 y) and adolescents with longer duration of HIV infection (median:18.5 y). (B) Frequencies of CD28^−^, CD57^+^, and CD57^+^CD28^−^ CD8^+^ T cells in perinatally HIV-1-infected children and adolescents. (C) Flow dot plots show CD8^+^ T cell surface expression of PD-1 and Tim-3 in perinatally HIV-1-infected children with shorter duration of HIV infection (median: 11.2 y) and adolescents with longer duration of HIV-1 infection (median: 18.5 y). (D) Frequencies of Tim-3^+^, PD-1^+^, and PD-1^+^ Tim-3^+^CD8^+^ T cells in perinatally HIV-1-infected children and adolescents. A significant increase in Tim-3^+^CD8^+^ T cell (*p = 0.045) population was observed with age. P values were obtained using two-tailed Mann-Whitney *U* test. Flow dot plots are representative of all subjects in respective groups.

**Figure 2:**
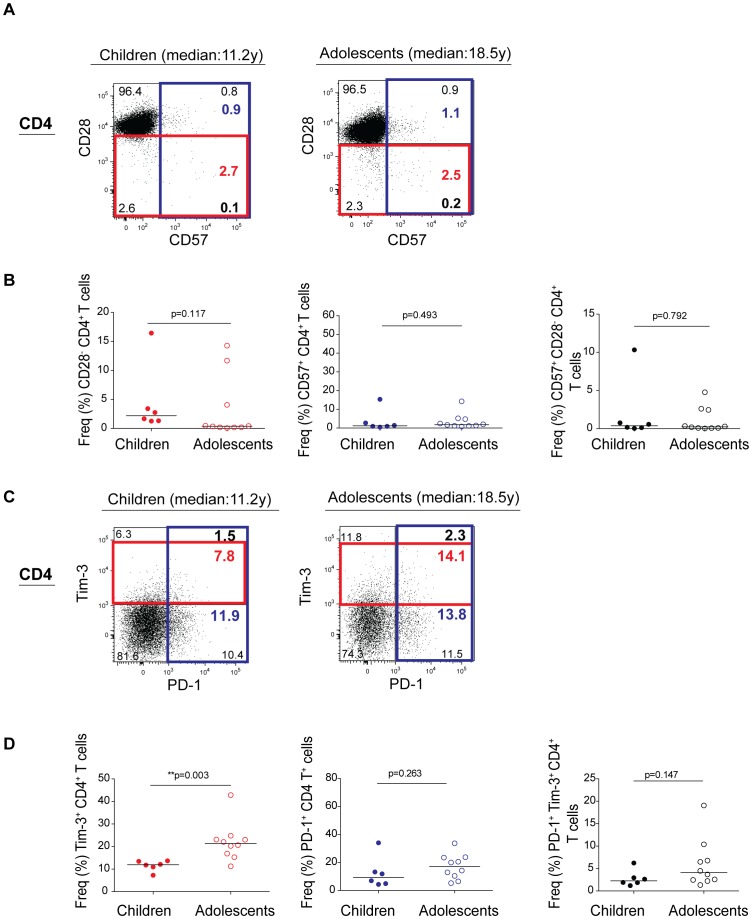
Expansion of Tim-3^+^ CD4^+^ T cells in perinatally HIV-1-infected adolescents. (A) Flow dot plots show CD4^+^ T cell surface expression of CD28 and CD57 in perinatally HIV-1-infected children with shorter duration of HIV-1 infection (median: 11.2 y) and adolescents with longer duration of HIV infection (median:18.5 y). (B) Frequencies of CD28^−^, CD57^+^, and CD57^+^CD28^−^ CD4^+^ T cells in perinatally HIV-1-infected children and adolescents. (C) Flow dot plots show CD4^+^ T cell surface expression of PD-1 and Tim-3 in perinatally HIV-1-infected children with shorter duration of HIV infection (median: 11.2 y) and adolescents with longer duration of HIV-1 infection (median: 18.5 y). (D) Frequencies of Tim-3^+^, PD-1^+^, and PD-1^+^ Tim-3^+^CD4^+^ T cells in perinatally HIV-1-infected children and adolescents. A significant increase in Tim-3^+^CD4^+^ T cell (**p = 0.003) population was observed with age. P values were obtained using two-tailed Mann-Whitney *U* test. Flow dot plots are representative of all subjects in respective groups.

T cell immunosenescence is characterized by the complete and permanent loss of CD28**^+^** T cells [16], [17] and elevated expression of CD57 on CD8**^+^** T cells [18]. To analyze the effect of the duration of HIV-1 infection and aging process on the T cell immune response, we assessed T cell differentiation and senescence (CD28^+^, CD57^+^, and CD57**^+^**CD28^−^) of both CD4**^+^** and CD8**^+^** T cells in two perinatally HIV-1 infected age groups on cART. We observed that CD8**^+^** and CD4**^+^** T cells did not show any difference in their senescence level between children and adolescents with shorter and longer duration of HIV-1 infection respectively, as observed by CD57^+^ (CD8: median 34.83%; IQR 22.84, 47.83 versus median 29.33%; IQR 21.29, 37.10; p = 0.367, figure 1A and B; CD4: median 1.18%; IQR 0.81, 5.87 versus median 1.84%; IQR 1.20, 4.88; p = 0.493, figure 2A and B), CD28^−^ (CD8: median 55.27%; IQR 36.42, 66.60 versus median 65.22%; IQR 54.66, 72.59; p = 0.263 figure 1A and B; CD4: median 2.21%; IQR 1.31, 6.67 versus median 0.38%; IQR 0.18, 5.97; p = 0.117, figure 2A and B) and CD57^+^CD28^−^ (CD8: median 31.55%; IQR 18.64, 39.52 versus median 24.61%; IQR 19.81, 31.88; p = 0.427, figure 1A and B; CD4: median 0.36%; IQR 0.10, 3.14 versus median 0.23%; IQR 0.07, 2.48; p = 0.792, figure 2A and B) levels.

### Expansion of Tim-3^+^CD4^+^ and CD8^+^ T cells in perinatally HIV-1 infected adolescents

In contrast to HIV-1 infection in adults, we did not observe any age related alterations in CD57 or CD28 T cell expression [18], [46]. We next assessed the expression of the inhibitory receptors, Tim-3 and PD-1. Compared to younger group, the frequency of Tim-3 in older group was increased in both CD8^+^ (median 18.35%; IQR 13.14, 20.95 versus median 23.93%; IQR 18.19, 32.94; *p = 0.045, figure 1C and D) and CD4^+^ (median 11.94%; IQR 10.04, 13.51 versus median 21.34%; IQR 16.47, 23.51; **p = 0.003, figure 2C and D) T cells. There was no significant difference in PD-1^+^CD8^+^ (median 40.05%; IQR 27.68, 55.80 versus median 36.75%; IQR 28.58, 51.71; p = 0.874, figure 1C and D) or PD-1^+^CD4^+^ (median 9.43%; IQR 4.82, 18.46 versus median 17.14%; IQR 9.48, 23.58; p = 0.263, figure 2C and D) and Tim-3^+^PD-1^+^CD8^+^ (median 5.55%; IQR 4.52, 6.67 versus median 6.23%; IQR 3.59, 12.07; p = 0.56, figure 1C and D) or Tim-3^+^PD-1^+^CD4^+^ T cell (median 2.26%; IQR 1.69, 3.76 versus median 4.08%; IQR 2.39, 7.67; p = 0.147, figure 2C and D) T cell population between groups. This suggests that Tim-3^+^ T cell expansion and not PD-1 is potentially driven by infection duration and possibly by age, and serves as a better marker for age related T cell dysfunction in the context of perinatal HIV-1 infection.

### Tim-3^+^CD8^+^ T cell expression and not PD-1^+^ is associated with HIV-1 viral load in adolescents with longer infection

**Figure 3:**
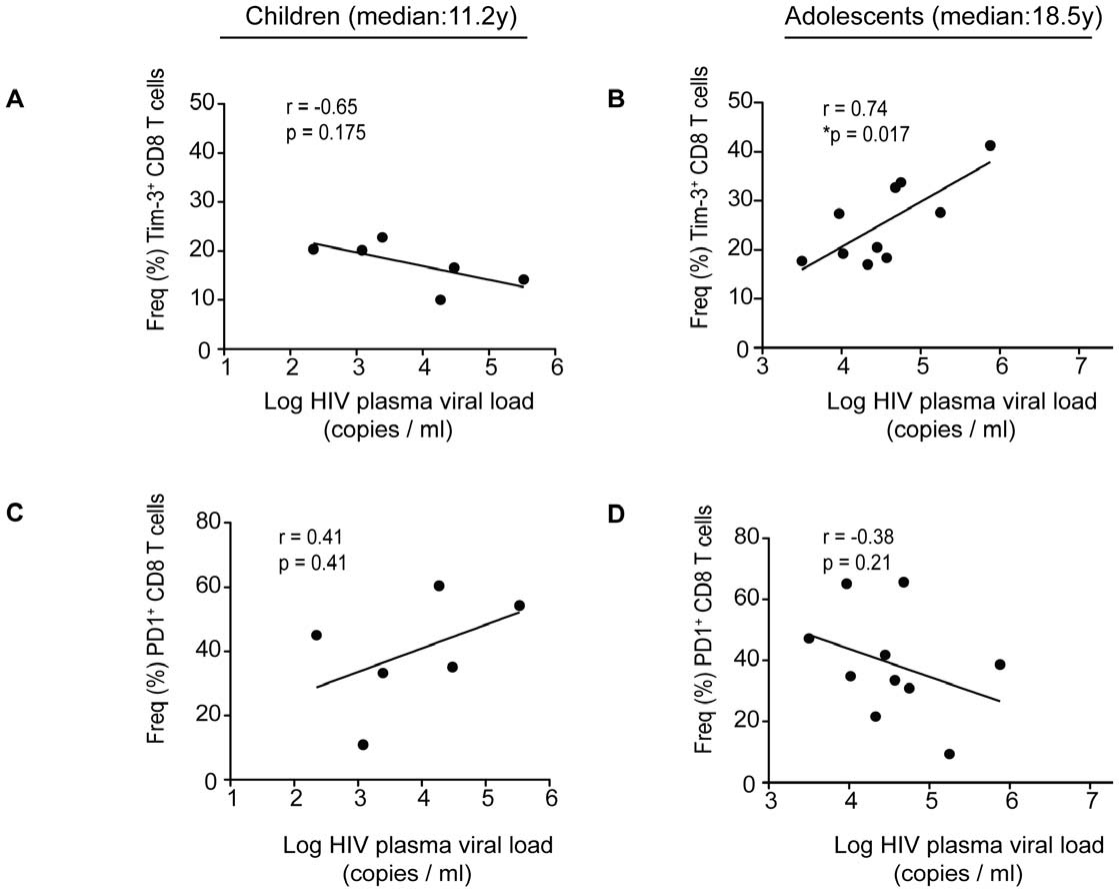
A positive correlation between Tim3^+^CD8^+^ T cells and HIV-1 plasma viral load (VL) in adolescents. Scatter plots showing correlation between HIV plasma viral load (VL) and Tim-3^+^CD8^+^ T cells (A and B), and PD-1^+^CD8^+^ T cells (C and D) in children (left) and adolescents (right) subjects. Tim-3^+^CD8^+^ T cells show a significant direct correlation with HIV-1 plasma viral load (VL) in adolescents (Spearman r = 0.74, *p = 0.017).

We and others previously observed that Tim-3 and PD-1 on CD8^+^ T cells are associated with HIV-1 viral load in adults [47]. We next determined whether the levels of Tim-3 or PD-1 on T cells were associated with HIV-1 plasma viral load. Interestingly, Tim-3^+^CD8^+^ T cells showed a significant direct association with HIV-1 plasma viral load in the adolescents (Figure 3B, *p = 0.017, *r* = 0.74), while no such association existed in the children (Figure 3A). PD-1^+^CD8^+^ T cells did not associate with HIV-1 plasma viral load in either of age groups (Figure 3C and D). Tim-3^+^CD4^+^ T cells did not show any association with HIV-1 plasma viral load in both children (Figure S1A) and adolescents (Figure S1B). However PD1^+^CD4^+^ T cells were directly associated with HIV-1 plasma viral load in children (Figure S1C, *p = 0.033, *r* = 0.88) but not in adolescents (Figure S1D).

### Infection duration and age related decline in CD8^+^ T cell activation in the adolescents with longer HIV infection

**Figure 4:**
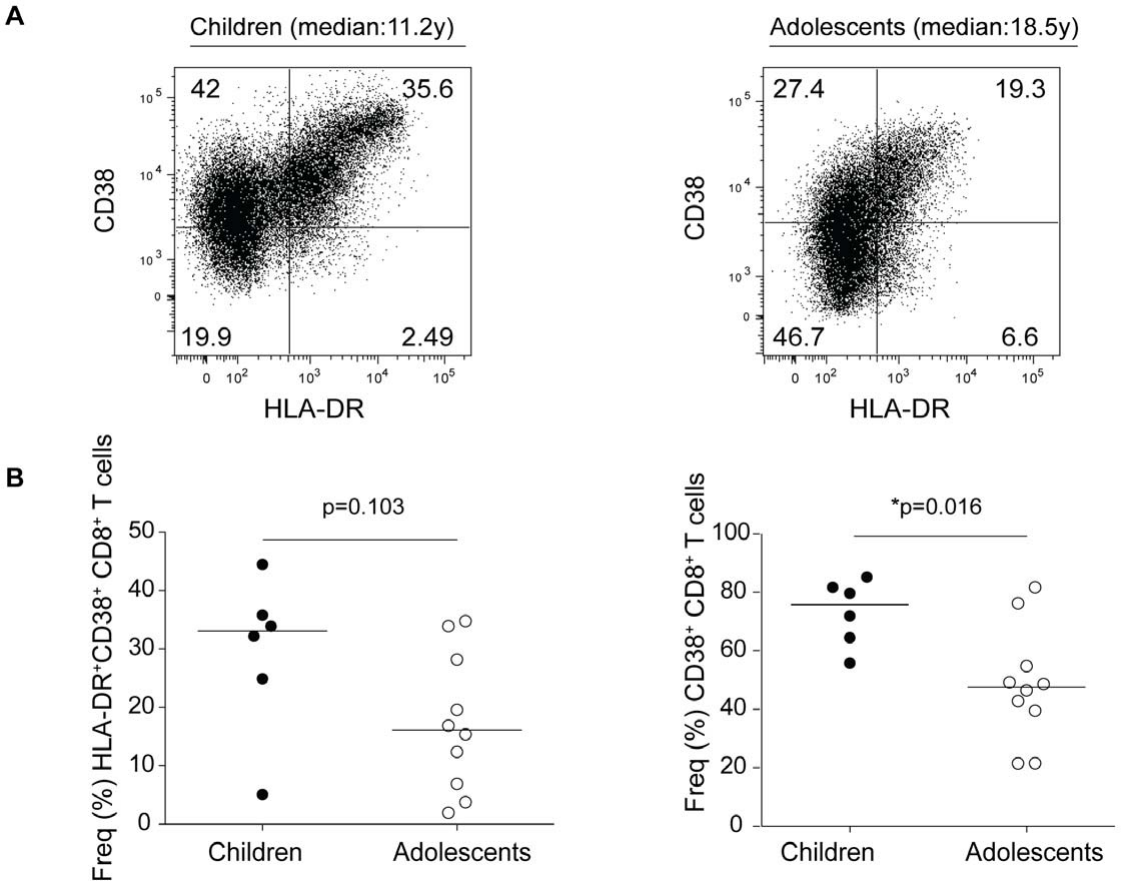
Infection duration and age related decrease in CD8^+^ T cell activation in adolescents. (A) Flow dot plots show CD8 surface expression of CD38 and HLA-DR in perinatally HIV-1 infected children (median: 11.2 y) and adolescents (median: 18.5 y). Dot plots are representative of all subjects in respective groups. (B) Frequencies of HLA-DR^+^CD38^+^CD8^+^ T cells and CD38^+^CD8^+^ T cells in perinatally HIV-1-infected children and adolescents. A significant decrease in CD38^+^CD8^+^ T cell (*p = 0.016) and a marginal decrease in HLA-DR^+^CD38^+^CD8^+^ T cell (p = 0.103) populations were observed with the duration of HIV infection. P values were obtained using two-tailed Mann-Whitney *U* test.

T cell activation, as defined by the expression of HLA-DR and CD38, is the marker of HIV-1 disease progression [37], [48], [49]. In perinatally HIV-1 infected children, CD38 expression in CD8^+^ T cells predicts virological failure despite antiretroviral therapy [50]. We determined next whether the levels of CD38 and HLA-DR or CD38 alone were associated with T cell activation. Given that perinatally HIV-1 children have a high HIV-1 load and poor adherence to cART, we hypothesized that perinatally HIV-1-infected children would show higher immune activation with advancing age. However, when we looked at treatment-experienced subjects from the two groups, we observed a decrease in immune activation in the adolescent group only, despite no significant change in viral load and CD4^+^ count in both groups. There was a marginal decrease in HLA-DR^+^CD38^+^CD8^+^ T cells in adolescents, while CD38^+^CD8^+^ T cells were significantly (*p = 0.016) decreased in adolescents (Figure 4A and B). The changes in immune activation were independent of HIV-1 plasma viral load (data not shown).

### PD-1^+^CD8^+^ T cells are associated with T cell activation but not viral load

**Figure 5:**
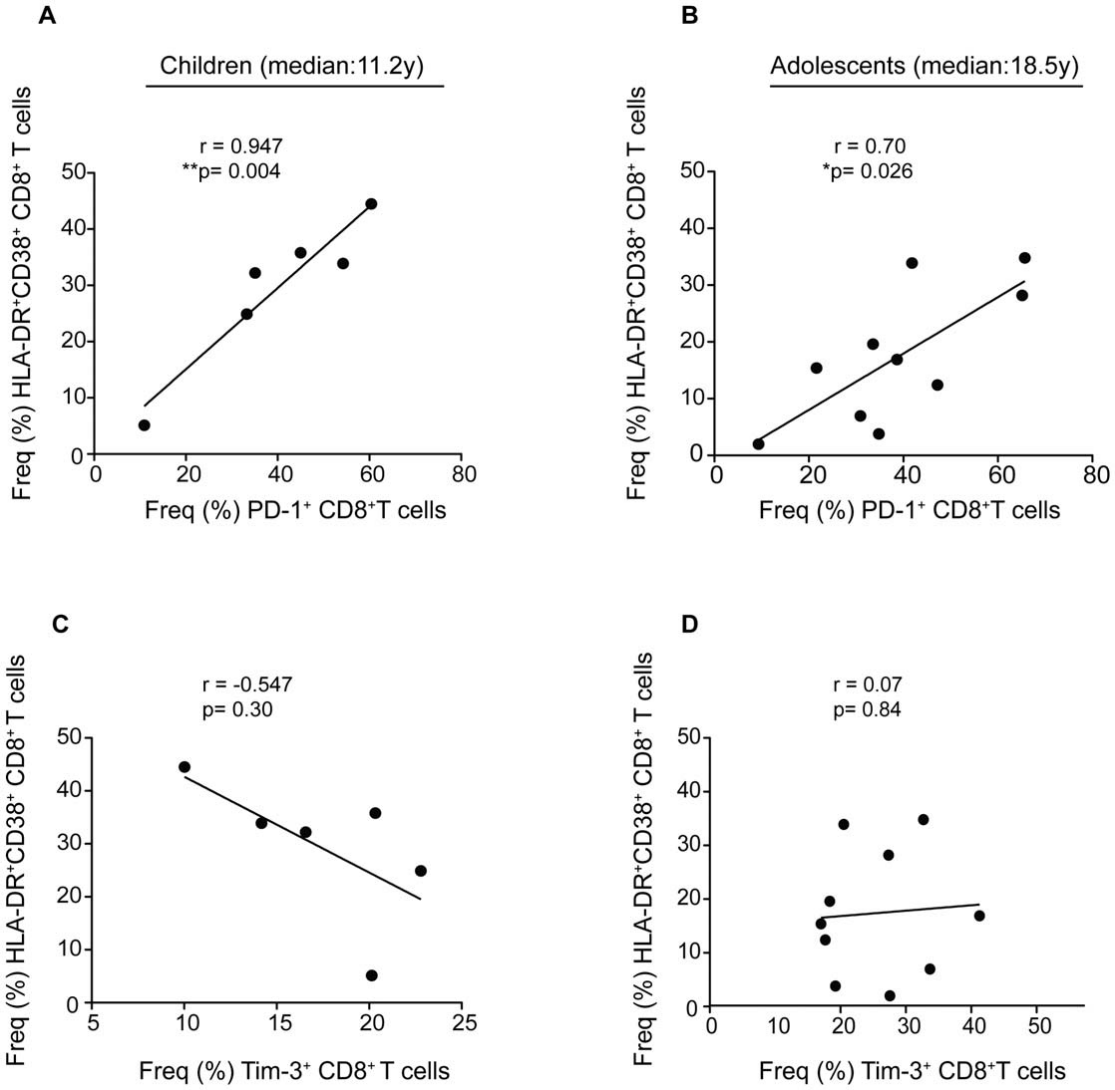
PD-1^+^CD8^+^ T cells have a strong significant correlation with immune activation in both age groups. Scatter plots showing a correlation between immune activation and PD-1^+^ or Tim3^+^CD8^+^ T cells. (A) and (B) show a significant strong correlation between HLA-DR^+^CD38^+^CD8^+^ T cell and PD-1^+^CD8^+^ T cells in children with shorter duration of HIV-1 infection (Spearman r = 0.947, **p = 0.004) and adolescents with longer duration of HIV-1 infection (Spearman r = 0.70, *p = 0.026) respectively. (C) and (D) show a correlation between HLA-DR^+^CD38^+^ CD8^+^ T cells and Tim-3^+^CD8^+^ T cells in both age groups.

We next assessed the relationship between T cell exhaustion and immune activation. Intriguingly, PD-1^+^CD8^+^ T cells had a direct strong significant association with HLA-DR^+^CD38^+^CD8^+^ T cells in children (r = 0.947, **p = 0.004) as well as in the adolescents (r = 0.70, *p = 0.026). This suggests that there is a possibility that PD-1 expression may be driven by immune activation (Figure 5A and B). Tim-3 did not show any association with immune activation in either group (Figure 5C and D).

## Discussion

Despite access to cART, children aging with HIV-1 infection are unable to control HIV-1 infection adequately [51]. Therefore, it is imperative to determine better correlates of protective immunity. This study shows that markers of T cell senescence do not appear to change with the duration of infection. However, there was an expansion of immune exhausted Tim-3^+^CD8^+^ T cells, but not PD-1^+^ T cells, in adolescents compared to younger children in the setting of high viremia. More importantly, these high levels of Tim-3^+^CD8^+^ T cells had a significant direct association with HIV-1 plasma viral load.

The expression of the inhibitory molecules Tim-3 and PD-1 on CD8 T cells has been associated with T cell exhaustion during chronic HIV-1 infection in adults. Their expression results in inhibition of T cell expansion and cytokine production [29], [30], [47], and associate with disease progression. A recent study has shown that PD-1 increased on T cells derived from older mice compared to younger mice [52], suggesting these exhaustion markers may contribute to age-related declines in immunity. Our data suggest that Tim-3 plays an important role in age-related T cell dysfunction in perinatal HIV-1 infection. By contrast, neither PD-1^+^ nor PD1^+^Tim-3^+^ T cells showed any difference in the two age groups in our study. While we did not observe differential PD-1 expression, this may be due to the small differences in age between the two groups in our study, or may reflect differences in the dynamics of PD-1 and Tim-3 expression.

T cell activation is undoubtedly the best marker of HIV-1 disease in adults. We and others have shown that HIV-1 viral load directly associates with T cell activation (HLA-DR^+^CD38^+^ or CD38^+^) in adults [53], [54], but this does not appear to occur in pediatric vertical infection which has also been confirmed by others [55]. Tim-3^+^ CD8^+^ T cells are not associated with T cell activation [30]. Our results are consistent with a recent report that shows a positive association between CD8^+^ T cell activation and the frequency of PD-1^+^CD8^+^ T cells in HIV-1 infected untreated younger children [56]. PD-1 may thus serve as a marker of T cell activation and our results build on this observation. Our previous study revealed that CD38 is associated with HIV-1 viral load [53]. We found that CD38 alone was lower in the adolescents with longer duration of HIV-1 infection despite both age groups having similar matched HIV-1 viral load.

T cell immunosenescence is characterized by the complete and permanent loss of CD28^+^ T cells and elevated expression of CD57 on CD8^+^ T cells [41], [43]. Compared to age-matched controls, HIV-1-infected adults have noticeable increase in CD28^−^ and CD57^+^ CD8^+^ T cell population [57], [58], [59]. In addition, CD28^−^CD8^+^ T cells have been shown to be significantly associated with the CD4^+^ T cell loss, and lymphocyte apoptosis in perinatally HIV-1-infected children [60]. In our present study, we expected an accumulation of CD57^+^CD28^−^ T cells in the adolescents with longer duration of infection. However, unlike adult HIV-1 infection, we did not observe any changes in these cell subsets in pediatric HIV-1 infection. These observations suggest that the previous published senescent markers (CD28, CD57) in adults may not reflect age related changes in perinatally HIV-1-infected children. Instead, Tim-3 may be a better marker of T cell immunosenescence in children and adolescents.

Due to the complexities in the management of perinatal HIV infection, and its accompanying specific treatment related issues, novel strategies are needed. Earlier initiation of cART follow by partial treatment interruption (PTI) is being reconsidered and envisioned to permit functional maturity of protective T-cell based immunity before introduction of a PTI regimen [61]. A recent study showed that adherence rates to antiretroviral therapy remained the same between PTI and continuous therapy (CT) [62]. Our previous work has shown that PTI was ineffective in controlling HIV-1 replication [63]. We propose that defining effective phenotypic profile of protective T cell immunity and reinvigorating T cell immunity by targeting age dependent pathways like Tim-3 may help induce effective anti-HIV-1 immune responses in children on either CT or PTI and be considered as an adjunctive therapy.

It is still debated whether T cell senescence and exhaustion are related or unrelated processes in compromised immunity. Detailed assessment of the signaling pathways that regulate T cell senescence and exhaustion suggest that they appear to be distinct processes [35]. Our study design did not permit the assessment of multiple age groups to determine the shifts in the profiles of the T cell differentiation and exhaustion markers over time in both HIV-1-infected and uninfected children. Therefore, further studies are needed to address these longitudinal changes in a large pediatric cohort. Our findings reveal the importance of the Tim-3 marker in pediatric HIV-1 infection and suggests that Tim-3 upregulation in the older age group was driven by HIV-1 viral replication, increased age, and longer duration of infection. The use of reagents targeting Tim-3 or its ligand could potentially reverse immunosenescence or exhaustion and other possible age-related complications. We have shown that blocking Tim-3-Tim-3L pathway either by addition of recombinant soluble Tim-3 (sTim-3) glycoprotein as a competitor for Tim-3 ligand(s) or using a blocking anti-Tim-3 mAB in vitro, increased expansion of antigen specific CD8^+^T cells and T cell proliferation responses [30]. Furthermore, we are now beginning to unravel the Tim-3 signaling pathway mediating T cell inhibition [64], [65], which could unveil potential targets that reverse Tim-3 mediated events. Applying these strategies to target Tim-3 in context of perinatal HIV-1 infection together with improved cART delivery strategies may therefore serve as a novel therapeutic to suppress viral replication and mitigate age-related complications offering further alternative treatment options in perinatal HIV-1 infection.

## Supporting Information

Figure S1 **A positive correlation between PD-1^+^CD4^+^ T cells and HIV plasma viral load (VL) in children.** Scatter plots showing correlation between HIV-1 plasma viral load (VL) and Tim-3^+^CD4^+^ T cells, and PD-1^+^CD4^+^ T cells in children (A and C) and adolescents (B and D) respectively. PD-1^+^CD4^+^ T cells show a significant direct correlation with HIV-1 plasma viral load (VL) in children with shorter duration of HIV infection (Spearman r = 0.88, *p = 0.033).
(TIF)
