# Supplementary figures and images for: Age-Related Expansion of Tim-3 Expressing T Cells in Vertically HIV-1 Infected Children
Source: PLoS One. 2012 Sep 24;7(9):e45733. doi: 10.1371/journal.pone.0045733 (PMC3454343; doi:10.1371/journal.pone.0045733)

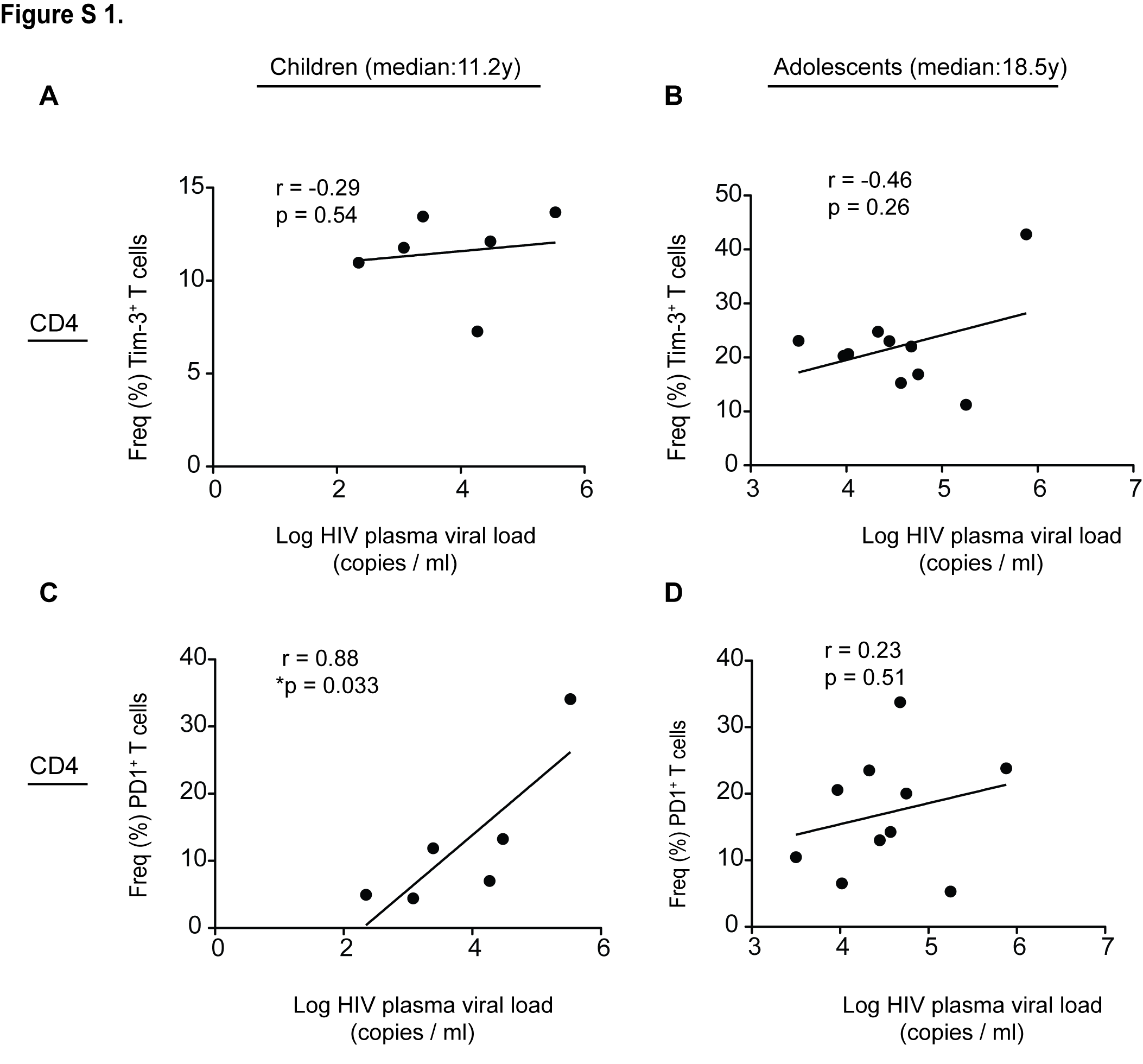

Supplement: Figure S1 — A positive correlation between PD-1+CD4+ T cells and HIV plasma viral load (VL) in children. Scatter plots showing correlation between HIV-1 plasma viral load (VL) and Tim-3+CD4+ T cells, and PD-1+CD4+ T cells in children (A and C) and adolescents (B and D) respectively. PD-1+CD4+ T cells show a significant direct correlation with HIV-1 plasma viral load (VL) in children with shorter duration of HIV infection (Spearman r = 0.88, *p = 0.033). (TIF) [file pone.0045733.s001.tif]
